# Diastolic dysfunction and the risk of end-stage kidney disease among patients with non-dialysis-dependent chronic kidney disease

**DOI:** 10.1007/s10554-025-03601-9

**Published:** 2026-01-09

**Authors:** Mathilde Zeuthen Pedersen, Kristoffer Grundtvig Skaarup, Nino Emanuel Landler, Flemming Javier Olsen, Jacob Christensen, Niklas Dyrby Johansen, Mats Christian Højbjerg Lassen, Christina Christoffersen, Ellen Linnea Freese Ballegaard, Ida Maria Hjelm Sørensen, Sasha Saurbrey Bjergfelt, Susanne Bro, Bo Feldt-Rasmussen, Ditte Hansen, Tor Biering-Sørensen

**Affiliations:** 1 Department of Cardiology, Copenhagen University Hospital - Herlev and Gentofte, Hellerup, Denmark; 2 Center for Translational Cardiology and Pragmatic Randomized Trials, Department of Biomedical Sciences, Faculty of Health and Medical Sciences, University of Copenhagen, Copenhagen, Denmark; 3 Department of Nephrology, Copenhagen University Hospital - Herlev and Gentofte, Herlev, Denmark; 4 Department of Nephrology, Copenhagen University Hospital - Rigshospitalet, Copenhagen, Denmark; 5 Department of Biomedical Sciences, Faculty of Health and Medical Sciences, University of Copenhagen, Copenhagen, Denmark; 6 Department of Clinical Biochemistry, Copenhagen University Hospital - Rigshospitalet, Copenhagen, Denmark; 7 Department of Clinical Medicine, Faculty of Health and Medical Sciences, University of Copenhagen, Copenhagen, Denmark; 8 Department of Medicine, Zealand University Hospital, Roskilde, Denmark

**Keywords:** Chronic cardiovascular-kidney disorder, Chronic kidney disease, Diastolic dysfunction, Echocardiography, Kidney failure

## Abstract

**Purpose:**

Left ventricular diastolic dysfunction (LVDD) is common in chronic kidney disease (CKD) and is suggested to be linked to worse renal prognosis. We investigated the associations between both the ratio of early mitral inflow velocity to early diastolic tissue velocity (E/e’) and LVDD grades with the risk of end-stage kidney disease (ESKD).

**Methods:**

This prospective cohort study included individuals with non-dialysis-dependent CKD from the nephrology outpatient clinics at Rigshospitalet and Herlev-Gentofte hospital, Copenhagen. All participants underwent a thorough, protocolized echocardiographic examination. The outcome was time to ESKD (defined as dialysis treatment or kidney transplantation).

**Results:**

A total of 731 participants were included. Mean age was 57 ± 13 years and 39% were female. During a median follow-up of 3.8 years (IQR: 3.3;4.5), 102 individuals reached ESKD. In multivariable Cox regression models, both increasing continuous E/e’ (HR = 1.09 [95%CI:1.03;1.15], P = 0.004, per 1 unit increase) and E/e’ > 14 (HR = 3.44 [95%CI:1.44;8.26], P = 0.005) (reference E/e’ < 8) were associated with ESKD, while E/e’ 8–14 was not. Compared with no or indeterminate LVDD, LVDD grades 2 or 3 were associated with ESKD (HR = 2.92 [95% CI:1.44;5.94], P = 0.003), while LVDD grade 1 was not. E/e’ > 14 and LVDD grade 2 or 3 demonstrated similar predictive accuracy with excellent specificity and negative predictive values, but poor positive predictive values and sensitivity.

**Conclusion:**

Prominent LVDD assessed by E/e’ and LVDD grades was independently associated with worse renal prognosis and had similar predictive accuracy in patients with non-dialysis dependent CKD.

**Graphical abstract:**

Diastolic dysfunction and the risk of ESKD among patients with non-dialysis-dependent CKD. The CPH-CKD study is a prospective cohort study of non-dialysis dependent CKD patients. All participants were systematically examined with a pre-defined protocolized echocardiography and followed for median 3.8 years- A total of 731 were included in the study sample and 102 developed ESKD. We found a significant, independent association between LVDD assessed by E/e’ and LVDD grades and worse renal prognosis in patients with non-dialysis dependent CKD. E/e’ and LVDD grading had similar predictive accuracy. Abbreviations: CKD (chronic kidney disease), ESKD (end-stage kidney disease), E/e’ (ratio of early mitral inflow velocity to early diastolic tissue velocity), LVDD (left ventricular diastolic dysfunction).

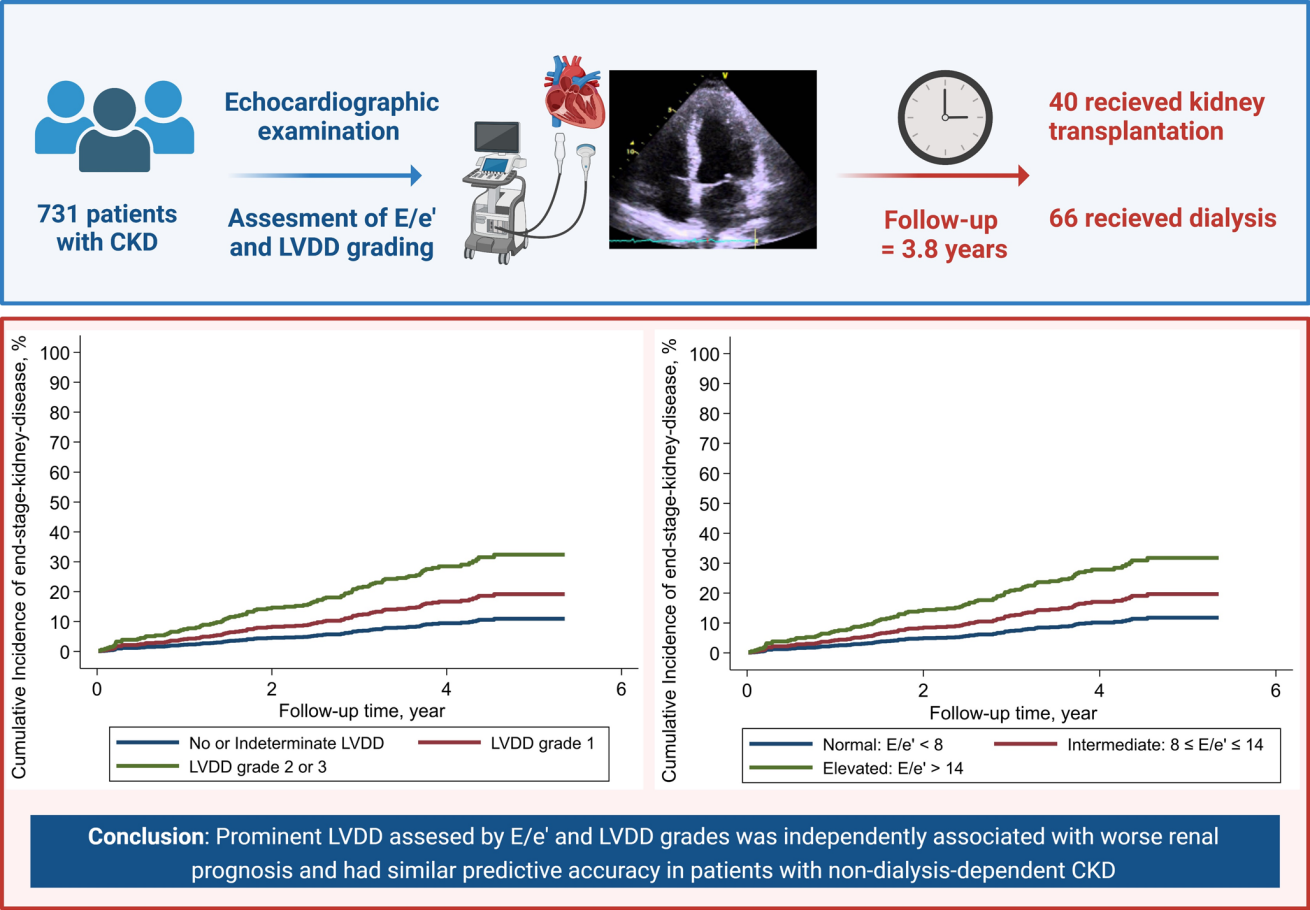

## Introduction

It is well-established that the heart and kidneys are closely interdependent where dysfunction in one may induce dysfunction in the other [1]. Several conceptual frameworks have been proposed to describe this interaction [2, 3]. Cardiac dysfunction is associated with a higher risk of death in patients with chronic kidney disease (CKD) [4], who subsequently have a 2–10 fold higher risk of dying from cardiovascular causes than the general population [5]. Additionally, cardiovascular disease in CKD patients is associated with an increased risk of end-stage kidney disease (ESKD), including dialysis or/and kidney transplantation [6].

Previous studies on the prevalence of abnormal cardiac structure and function assessed by echocardiography in patients with CKD have established that patients with CKD frequently suffer from diastolic dysfunction [6, 7]. This is likely explained by the common concomitant conditions of CKD including hypertension, elevated central venous pressure (CVP) and increased fluid volume, which increase afterload and preload, collectively leading to elevated left ventricular (LV) filling pressures, LV hypertrophy, and abnormal myocardial relaxation [2, 3, 8–10].

The ratio of early diastolic mitral inflow velocity to early diastolic tissue velocity (E/e’) measured by tissue Doppler echocardiography, a surrogate marker of LV filling pressures, is a rapidly measured and feasible echocardiographic parameter used to evaluate LV diastolic dysfunction (LVDD) [11, 12]. Elevated E/e’ is a frequent finding in patients with CKD and is one of the earliest echocardiographic parameters to improve after kidney transplantation, further emphasizing the close interaction between cardiac and kidney function [9]. Additionally, E/e’ has been associated with both cardiovascular morbidity and mortality in CKD [4, 13] and, more recently, among Asian CKD patients in the KNOW-CKD study, linked to worse renal prognosis i.e. dialysis or kidney transplantation [11]. However, comprehensive assessment of LVDD requires measurement of additional echocardiographic markers [14]. The association between LVDD as graded by current guidelines and progression to ESKD is currently unknown.

In addition, whether the association between E/e’ and renal prognosis found in the KNOW-CKD study translates to other ethnicities has not yet been studied.

The aim of this study was to investigate the relationship between E/e’ and LVDD grades and risk of end-stage kidney disease in non-dialysis-dependent CKD patients.

## Methods

### Study population

The CPH-CKD ECHO study is a Danish two-center prospective cohort, which consecutively included patients with CKD receiving outpatient nephrology care in the period September 2015 to August 2018 from the departments of Nephrology at Copenhagen University Hospitals - Rigshospitalet and Herlev-Gentofte. Inclusion criteria were patients aged 30 to 75 years with CKD and estimated glomerular filtration rate (eGFR) stages G1-G5 pre-dialysis. Exclusion criteria were (1) previous kidney transplantation, (2) pregnancy, (3) intellectual disability, dementia or psychosis, (4) active malignancy, (5) withdrawn informed consent or unwilling to attend the necessary examinations. All participants gave written informed consent. The CPH-CKD ECHO study was performed in agreement with the 2nd Declaration of Helsinki and approved by a local ethics committee.

### Baseline characteristics and outcome

Baseline examinations were performed as previously described [8]. In brief, all participants were interviewed and had their electronic medical records reviewed to obtain information on baseline characteristics including symptoms of cardiovascular and renal disease, comorbidities, and medication use. All participants underwent a thorough clinical examinational to obtain clinical parameters including body mass index (BMI) and blood pressure. Venous blood samples and urine samples were collected and analyzed. Urine albumin-to-creatinine ratio (UACR) was calculated. eGFR was calculated using the CKD Epidemiology Collaboration (CKD-EPI) formula [15]. CKD stages were defined according to the 2012 Kidney Disease: Improving Global Outcomes (KDIGO) guidelines [16].

### Outcome

The outcome was time to ESKD, defined as a composite of dialysis or kidney transplantation. Follow-up was performed through manual electronic health record review. Follow-up was 100%.

### Echocardiography

Echocardiography was performed with GE Vivid E9 ultrasound machines (GE Healthcare, Horten, Norway) by experienced sonographers. All individuals were examined with echocardiography following a predefined comprehensive protocol while positioned in left lateral decubitus position. All projections were recorded as three consecutive cardiac cycles. A single investigator analyzed the echocardiographic images blinded to clinical and outcome data. Echocardiographic analysis was performed with the commercially available software EchoPAC v.202 (GE Healthcare, Horten, Norway).

Echocardiographic measurements were performed and defined according to current guidelines [17]. The methods have previously been described in detail [8]. In brief, LV chamber dimensions were measured in the parasternal long-axis view at the level of the mitral valve leaflet tips in end-diastole. LV mass was calculated using the American Society of Echocardiography (ASE) formular [17]. Using the Simpsons biplane method LV ejection fraction was determined from the apical 4-chamber and 2-chamber views. Peak early mitral inflow velocity (E) was measured with pulsed-wave Doppler imaging at the tip of the mitral valve leaflets in the apical 4-chamber view. Utilizing pulsed-wave tissue Doppler imaging with sample areas at the septal and lateral walls of the mitral annulus, the mean diastolic peak early tissue velocity (e’) was measured and E/e’ was calculated. Definition and grading of LVDD (no LVDD, indeterminate, LVDD grade 1, 2, and 3) was performed as previously described [8], in accordance with the 2016 ASE and European Association of Cardiovascular Imaging (EACVI) guidelines [14].

E/e’ was stratified into three groups: normal (E/e’ < 8), intermediate (E/e’ 8–14), and elevated (E/e’ > 14). The same applied for LVDD grades, where the three groups represent no LVDD (no or indeterminate LVDD), intermediate LVDD (LVDD grade 1), and prominent LVDD (LVDD grade 2 or 3).

### Statistics

All analyses were performed using STATA SE version 17.0. A P-value < 0.05 was defined as statistically significant. Gaussian distributed variables are displayed as means ± standard deviation and compared using two-sample t-tests. Non-Gaussian distributed variables are displayed as medians with interquartile range and compared using Kruskal-Wallis tests. Finally, categorical variables are listed as frequencies with percentages and compared with Chi-square tests. To assess the association between LVDD parameters and time to the composite outcome, Cox proportional hazard models were utilized. Both univariable and multivariable models were fitted. The multivariable model included age, sex, eGFR, diabetes, BMI, atrial fibrillation, UACR, systolic blood pressure, heart rate, history of coronary artery disease, and LV ejection fraction. To illustrate the relationship between continuous E/e’ and incidence rate of the outcome, restricted cubic spline curves were created. Tests for linearity and non-linearity were carried out, and number of knots was chosen according to lowest Akaike information criterion. Cumulative failure rate during follow-up according to the three categories of E/e’ and LVDD grades were estimated with cumulative incidence curves (with all-cause mortality as competing event). Finally, sensitivity, specificity, and negative and positive predictive values for E/e’ > 14 vs E/e’ ≤ 14 and LVDD grade 2 or 3 vs. LVDD grade 1, indeterminate, or no LVDD in relation to the composite outcome were calculated.

## Results

### Demographic and clinical data

A total of 731 CKD patients were included in the final study sample. Mean age was 57 ± 13 years and 287 (39%) were female. Median eGFR was 43 mL/min/1.73 m² (IQR: 28;62), and 260 (36%) had UACR > 300. Median E/e’ was 7.8 (IQR: 6.4; 9.5) and 362 (50%) had no LVDD, 5 (1%) had indeterminate LVDD, 316 (43%) had LVDD grade 1, 41 (6%) had LVDD grade 2, and 7 (1%) had LVDD grade 3. Baseline data stratified by E/e’ > 14 are listed in Table 1. Patients with E/e’ >14 were older and more likely to have diabetes, hypertension, and coronary artery disease.

**Table 1:** Baseline characteristics of all and according to E/e’

Clinical characteristics	Total *n*: 731	E/e’ ≤ 14 *n*: 700	E/e’ > 14 *n*: 31	*P*-value
Age, years	57.11 (12.79)	56.59 (12.78)	68.76 (5.00)	< 0.001
Male sex, n (%)	444 (60.7)	426 (60.9)	18 (58.1)	0.76
Systolic blood pressure, mmHg	131.56 (17.32)	131.03 (16.73)	143.55 (24.99)	< 0.001
Diastolic blood pressure, mmHg	81.49 (10.65)	81.72 (10.42)	76.42 (14.20)	0.01
Heart rate, beats/minute	72.88 (13.13)	72.99 (12.95)	70.55 (16.92)	0.31
*Smoking status, n (%)*				0.65
Never	303 (41.6)	291 (41.7)	12 (38.7)	
Active	127 (17.4)	123 (17.6)	4 (12.9)	
Former	299 (41.0)	284 (40.7)	15 (48.4)	
Body mass index, kg/m²	28.27 (5.81)	28.13 (5.64)	31.50 (8.18)	< 0.001
*Medical history*				
Hypertension, n (%)	619 (84.7)	589 (84.1)	30 (96.8)	0.06
Diabetes, n (%)	136 (18.6)	121 (17.3)	15 (48.4)	< 0.001
Coronary artery disease, n (%)	36 (4.9)	31 (4.4)	5 (16.1)	< 0.001
Previous myocardial infarction, n (%)	15 (2.1)	11 (1.6)	4 (12.9)	< 0.001
Previous coronary artery bypass grafting, n (%)	7 (1.0)	5 (0.7)	2 (6.5)	< 0.001
Atrial fibrillation, n (%)	46 (6.3)	44 (6.3)	2 (6.5)	0.97
Cerebrovascular disease, n (%)	63 (8.6)	57 (8.1)	6 (19.4)	0.03
Peripheral artery disease, n (%)	29 (4.0)	24 (3.4)	5 (16.7)	< 0.001
*Medication*				
Antihypertensive treatment, n (%)	572 (78.2)	542 (77.4)	30 (96.8)	0.01
ACEI/ARB, n (%)	471 (64.4)	450 (64.3)	21 (67.7)	0.69
Beta blockers, n (%)	204 (27.9)	186 (26.6)	18 (58.1)	< 0.001
Calcium channel blockers, n (%)	281 (38.4)	268 (38.3)	13 (41.9)	0.68
Aldosterone antagonist, n (%)	33 (4.5)	32 (4.6)	1 (3.2)	0.72
Diuretics, n (%)	396 (54.2)	371 (53.0)	25 (80.6)	< 0.001
*Biochemistry*				
Cholesterol, mmol/L	5.18 (1.24)	5.20 (1.23)	4.69 (1.23)	0.02
eGFR, mL/min/1.73 m²	43 [28;62]	43 [29;64]	32 [22;43]	< 0.001
Urine Albumin/Creatinine ratio mg/g	131.00 [22.00;678.50]	128.00 [22.00;653.00]	230.50 [24.75;1097.25]	0.17
*Echocardiography*				
E/e’	7.81 [6.40;9.49]	7.70 [6.32;9.16]	16.65 [15.15;20.79]	< 0.001
LVEF, %	59.16 (6.36)	59.28 (6.25)	56.32 (8.04)	0.01
Grade of LVDD, n (%)				< 0.001
Normal diastolic function	362 (49.5)	360 (51.4)	2 (6.5)	
Grade 1	316 (43.2)	308 (44.0)	8 (25.8)	
Grade 2	41 (5.6)	21 (3.0)	20 (64.5)	
Grade 3	7 (1.0)	6 (0.9)	1 (3.2)	
Indeterminate	5 (0.7)	5 (0.7)	0 (0.0)	

ACEI (angiotensin converting enzyme inhibitors), ARB (angiotensin receptor blocker), E/e’ (ratio of early mitral inflow velocity to early diastolic tissue velocity), eGFR (estimated glomerular filtration rate), LVEF (left ventricular ejection fraction)

### Follow-up

Median follow-up time was 3.8 years (IQR: 3.3–4.5), and follow-up was 100%. In total, 102 patients met the composite outcome; 66 were treated with dialysis, and 40 received kidney transplants. The proportion of patients with events increased as LVDD worsened in both the E/e’ and LVDD grade categories (E/e’ < 8 = 11.3%, E/e’ 8–14 = 14.8%, E/e’ >14 = 38.7%, and no LVDD or indeterminate = 11.2%, LVDD grade 1 = 13.3%, LVDD grade 2 or 3 = 40%).

### Relationship between LVDD parameters and risk of end-stage kidney disease

**Fig. 1:**
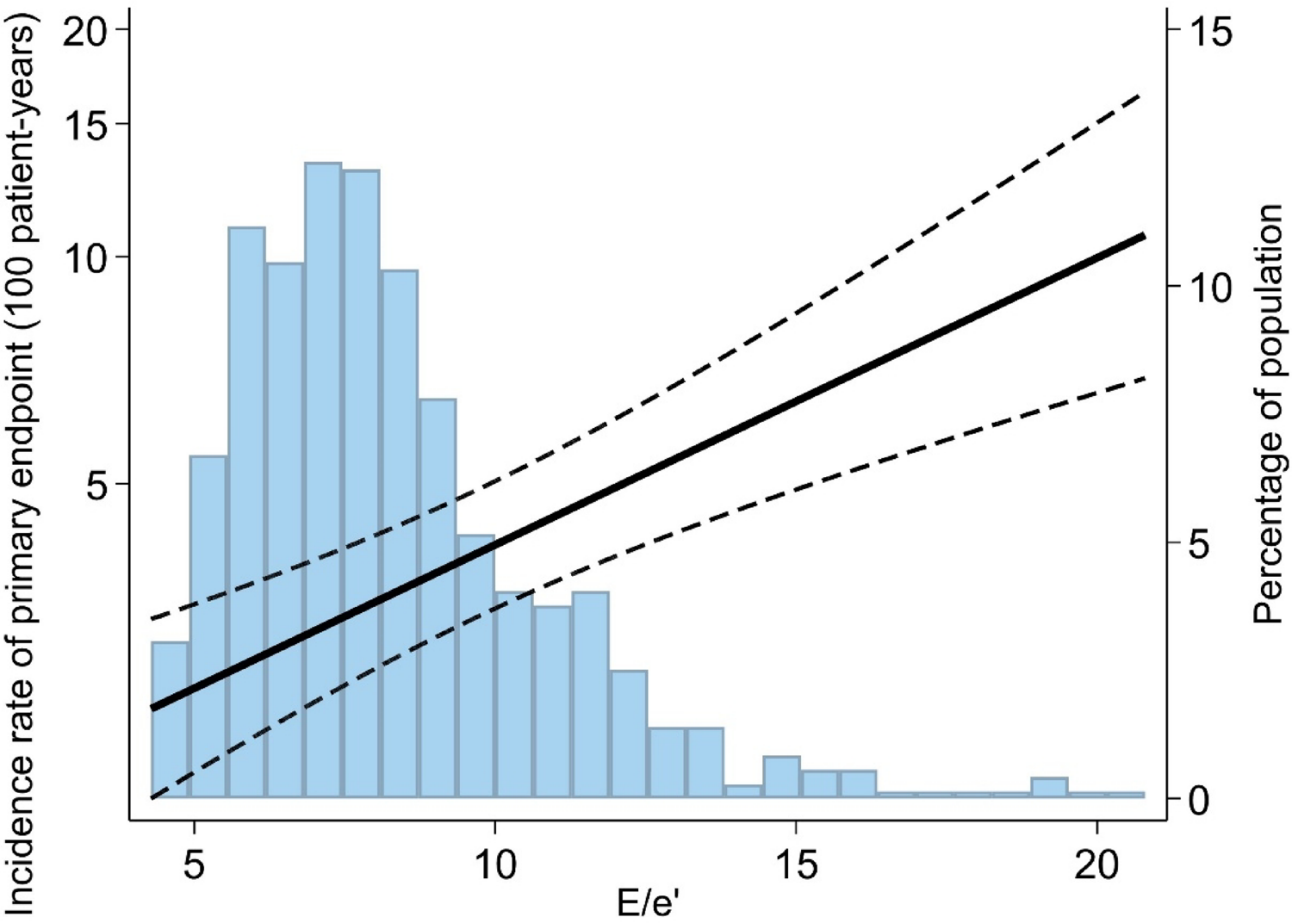
The incidence rate of dialysis and/or kidney transplantation according to E/e’ Caption: Restricted cubic spline curves illustrating incidence rate per 100 patient-years according to E/e’. Abbreviation: E/e’ (ratio of early mitral inflow velocity to early diastolic tissue velocity)

Results from Cox regression models are listed in Table 2. Increasing continuous E/e’ and E/e’ >14 (reference: E/e’ < 8) were significantly associated with increased risk of the composite outcome in univariable Cox regression, while E/e’ in the intermediate range (8–14) was not. Continuous E/e’ (HR = 1.09 [95% CI:1.03;1.15], P = 0.004, per 1 unit increase) remained significantly associated with an elevated risk of the composite outcome after multivariable adjustment. In contrast, LVEF was not associated with outcome in the same model (HR = 1.00, [95% CI: 0.97; 1.03], P = 0.92, per 1% decrease). Continuous E/e’ displayed a linear relationship with the incidence rate of the composite outcome, which is depicted in Fig. 1 in a restricted cubic spline curve. In the multivariable Cox model, E/e’ > 14 remained significantly associated with worse renal outcome (HR = 3.44 [95% CI:1.44; 8.26], P = 0.005), while E/e’ 8–14 did not (HR = 1.25 [95% CI:0.75;2.06], *P* = 0.39).

**Table 2:** Univariable and multivariable Cox regression models

	Univariable regression			Multivariable regression*		
	Hazard ratio	95% CI	*P*-value	Hazard ratio	95% CI	*P*-value
*E/e’*						
E/e’, per 1 increase	1.09	1.05;1.13	< 0.001	1.09	1.03;1.15	0.004
E/e’ < 8 (reference)	1.00	-	-	-	-	-
8 ≤ E/e ≤ 14	1.41	0.93;2.13	0.11	1.25	0.75;2.06	0.39
E/e’ > 14	4.29	2.26;8.13	< 0.001	3.44	1.44;8.26	0.005
*LVDD grades*						
No LVDD or indeterminate (reference)	1.00	-	-	1.00	-	-
LVDD grade 1	1.21	0.79;1.86	0.38	1.41	0.69;1.92	0.60
LVDD grade 2 or 3	4.55	2.64;7.85	< 0.001	2.92	1.44;5.94	0.003

*Multivariable regression included age, sex, eGFR, diabetes, body mass index, urine albumin to creatinine ratio, atrial fibrillation, systolic blood pressure, heart rate, coronary artery disease, and left ventricular ejection fraction

CI (confidence interval), E/e’ (ratio of early mitral inflow velocity to early diastolic tissue velocity), eGFR (estimated glomerular filtration rate), LVDD (left ventricular diastolic dysfunction)

**Fig. 2A and B:**
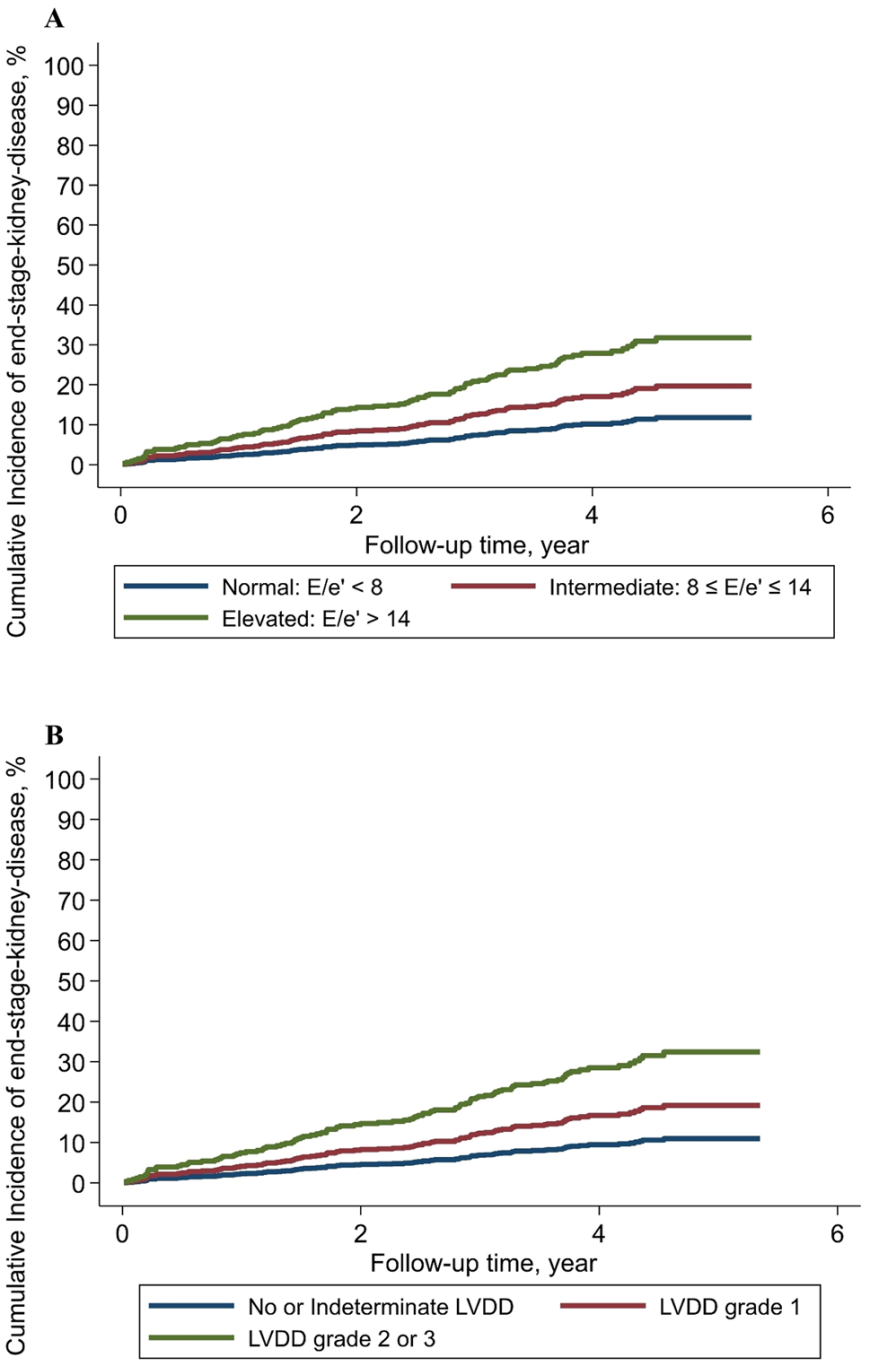
Cumulative incidence of dialysis and/or kidney transplantation. Cumulative incidence rate of dialysis or kidney transplantation during follow-up according to normal: E/e’ < 8, intermediate: 8 ≤ E/e ≤ 14, and elevated: E/e’ >14 in Fig. 4 A and no or indeterminate LVDD, LVDD grade 1, and LVDD grade 2 or 3 in Fig. 4B. Abbreviations: E/e’ (ratio of early mitral inflow velocity to early diastolic tissue velocity), LVDD (left ventricular diastolic dysfunction)

A similar pattern was observed when evaluating LVDD grades. In univariable Cox regression models, LVDD grade 2 or 3 was associated with outcome compared with no or indeterminate LVDD, while LVDD grade 1 was not (Table 2). Following multivariable adjustment, LVDD grade 2 or 3 remained independently associated with increased risk of progression to ESKD (HR = 2.92 [95% CI:1.44;5.94] P = 0.003). The cumulative incidence of kidney transplantation or dialysis during follow-up according to E/e’ groups and LVDD grades is illustrated in Fig. 2A and B, respectively.

### Predictive accuracy of E/e’ and LVDD grades

Sensitivity, specificity, negative, and positive predictive values for E/e’ > 14 vs E/e’ ≤ 14 and LVDD grade 2 or 3 vs LVDD grade < 2 regarding treatment with dialysis or kidney transplantation are listed in Table 3 according to various follow-up (1, 2, and 3 years and full). E/e’ > 14 and LVDD grade 2 or 3 demonstrated similar predictive accuracy across follow-up times. Both had excellent specificity (96.1–97.0% for E/e’ > 14 and 94.3–95.2% for LVDD grade 2 or 3) and negative predictive values (87.1–96.8% and 87.8–97.3%), but only poor sensitivity (11.8–12.2% and 18.6–28.0%) and poor to modest positive predictive values (10.0–38.0% and 14.9–39.6%).

**Table 3:** Predictive accuracy of E/e’ and LVDD

	E/e’ > 14*	LVDD grade 2 or 3**
*Full follow-up (n = 731)*		
Sensitivity (95% CI) Specificity (95% CI)	11.8% (6.23;19.6%) 97.0% (95.3;98.2%)	18.6% (11.6;27.6%) 95.4% (93.4;96.9%)
Positive predictive value (95% CI) Negative predictive value (95% CI)	38.0% (21.8;57.8%) 87.1% (84.4;89.5%)	39.6% (25.8;54.7%) 87.8% (85.2;90.2%)
*1 year follow-up (n = 724)*		
Sensitivity (95% CI) Specificity (95% CI)	12.0% (2.6;31.2%) 96.1% (94.4;97.4%)	28.0% (12.1;49.4%) 94.3% (92.3;95.9%)
Positive predictive value (95% CI) Negative predictive value (95% CI)	10.0% (2.1;26.5%) 96.8% (95.2;98.0%)	14.9% (6.2;28.3%) 97.3% (95.8;98.4%)
*2-year follow-up (n = 715)*		
Sensitivity (95% CI) Specificity (95% CI)	12.0% (4.5;24.3%) 96.4% (94.7;97.7%)	24.0% (13.1;38.2%) 95.0% (93.1;96.6%)
Positive predictive value (95% CI) Negative predictive value (95% CI)	20.0% (7.7;38.6%) 93.6% (91.5;95.3%)	26.7% (14.6;41.9%) 94.3% (92.3;96.0%)
*3-year follow-up (n = 698)*		
Sensitivity (95% CI) Specificity (95% CI)	12.2% (5.71;21.8%) 96.8% (95.1;98.0%)	20.3% (11.8;31.2%) 95.2% (93.2;96.7%)
Positive predictive value (95% CI) Negative predictive value (95% CI)	31.0% (15.3;50.8%) 90.3% (87.8;92.4%)	33.3% (20.0;49.0%) 91.0% (88.5:93.1%)

*Compared with E/e’ < 14

**Compared with LVDD grade 1, indeterminate or no LVDD

CI (confidence interval), E/e’ (ratio of early mitral inflow velocity to early diastolic tissue velocity), LVDD (left ventricular diastolic dysfunction)

## Discussion

In this analysis from the large, prospective, two-center CPH-CKD ECHO cohort including 731 patients with non-dialysis-dependent CKD, we found that LVDD as evaluated by both E/e’ alone and graded according to current guidelines was associated with an increased risk of ESKD. Following multivariable adjustment, continuous E/e’ and E/e’ > 14 were independently associated with worse renal outcomes, whereas E/e’ 8–14 was not. Similarly, LVDD grade 2 and 3 also remained independently associated with renal outcomes, while LVDD grade 1 was not. E/e’ > 14 and LVDD grade 2 and 3 had similar predictive accuracy with excellent specificity and negative predictive values.

Other studies have previously addressed the topic regarding diastolic function and renal prognosis [11, 18–20]. Most of these have been limited to patients with CKD stage G3 or above [18, 19], have investigated limited or outdated diastolic measures [18], and used composite endpoints that often included both “hard” endpoints (i.e. initiation of dialysis or renal transplantation) and renal function decline by changes in eGFR or creatinine [11, 18, 20], which may however be less clinically robust.

Borrelli et al.^20^ previously investigated the association between LVDD classification by the guideline-recommended grading system and renal prognosis in patients with G1-G5 non-dialysis CKD (n: 140), but there were generally few events and the majority of events were eGFR changes. Additionally, that study did not relate individual diastolic measures to renal outcomes.

The largest comparable study is the prospective Korean KNOW-CKD study [11], which recently reported an association between septal E/e’ and increased risk of renal events, thereby suggesting E/e’ as a novel risk factor in patients with CKD. The KNOW-CKD study was conducted from 2011 to 2015, enrolling 2,238 patients with an ongoing follow-up and considered a > 50% decrease in eGFR rate from baseline, doubling of serum creatinine or dialysis and/or kidney transplantation as renal events.

A major strength of our study is that it is both one of the first and the largest European study to investigate this association. Furthermore, our substantial sample was consecutively included, examined by state-of-the-art echocardiographic methods, and the echocardiographic evaluators were blinded to all patient information. The KNOW-CKD study sought to investigate the association using solely septal E/e’, an important consideration since the utilization of an averaged e’ provides a more comprehensive and global evaluation of E/e’. The Korean investigators emphasized the need for further investigation using a complete evaluation of LVDD, which requires additional echocardiographic markers besides E/e’ alone [21]. We attempted to take this into account by considering both septal and lateral e’ velocities for calculating E/e’, tricuspid regurgitation velocity, and left atrial volume according to the ASE/EACVI guidelines when evaluating participants for LVDD [14]. We found both E/e’ and LVDD grading to be independently associated with worse renal prognosis, which aligns with the findings on E/e’ by the KNOW-CKD study. Ultimately, due to the different genetic and environmental factors of the individuals in each study, E/e’ could potentially be a more generalizable measurement.

The similar predictive values of E/e’ >14 compared with LVDD grades 2 and 3 suggest that E/e’ as a single measure may be a viable alternative to the LVDD grading system. The complexity of the LVDD grading system has shown to affect its dissemination as it is infrequently reported [22], whereas the E/e’ is a simple, fast, and easily comprehensible measure, that is closely related to filling pressure [14]. Furthermore, both E/e’ and LVDD grading may be most useful in identifying patients with CKD with no or low risk of developing renal outcomes since elevated E/e’ and LVDD grade 2 and 3 demonstrated excellent negative predictive value, whereas the positive predictive value and sensitivity were poor. These findings are worth considering when or if a patient with CKD is having echocardiography performed. Our findings do not imply that screening for LVDD should be performed in all CKD patients since neither clinical benefit nor cost-effectiveness has been demonstrated in a randomized setting. We acknowledge that measurements of kidney function, namely UACR and eGFR, are primary predictors of disease progression in CKD; however, the associations between E/e’ and LVDD grades and progression towards worse renal outcome found in this study were independent of UACR and eGFR. While the focus of this study was on the use of E/e’, it would be interesting to investigate whether this measure could be used in concert with other potential biomarkers. Accordingly, future studies could consider incorporating these and other emerging renal and cardiac biomarkers reflecting shared pathophysiological pathways between the heart and kidneys, such as markers of inflammation, oxidative stress, and neurohormonal activation. Prospective studies and trials are, however, still needed to evaluate whether such markers would improve clinical risk schemes to guide closer monitoring for renal deterioration.

There are several potential pathophysiological mechanisms behind the observed link between LVDD and renal prognosis. A possible explanation lies in the cardio-renal interaction outlined by the chronic cardiovascular-kidney disorder (CCKD) [23], in which shared risk factors (i.e. hypertension, diabetes) can lead to diastolic dysfunction either by themselves or in combination with pre- and afterload changes induced by renal dysfunction through activation of the renin-angiotensin-aldosterone-system. A patient with both CKD and LVDD could consequently be at risk of entering a vicious circle: higher degrees of diastolic dysfunction could lead to reduced renal perfusion pressure due to elevated CVP, further activating the renin-angiotensin-aldosterone-system leading to sodium and fluid retention. This secondarily causes increased afterload and cardiac fibrosis leading to further increase of LV filling pressures and, ultimately, further increase of CVP, hence worsening of renal perfusion. This could be aggravated by reduced cardiac output and stroke volume due to increased afterload [2, 3, 10]. Therefore, LVDD could potentially be a sign of unfavorable renal perfusion conditions, which cannot be assessed by routine eGFR or UACR measurements alone.

### Limitations

A degree of selection bias is likely present in our study as inclusion was limited to patients with CKD in an outpatient hospital setting. Thus, our cohort may not fully represent patients with CKD managed exclusively in primary care or those who remain undiagnosed, which potentially limits the external validity and generalizability of our findings. In addition, the study was conducted in a European hospital-based population, and the findings may therefore not be generalizable to patients with CKD in non-European populations or populations with different demographic or clinical characteristics. Consequently, studies with more diverse populations are still needed. We cannot exclude the possibility of residual confounding in that we may have been unable to sufficiently adjust for baseline differences between patients with different levels of LVDD. Finally, we were unable to include other renal biomarkers such as cystatin C and neutrophil gelatinase-associated lipocalin in our models.

## Conclusion

In a large, prospective cohort study, we found a significant, independent association between LVDD assessed by E/e’ and LVDD grades and worse renal prognosis in patients with non-dialysis dependent CKD. E/e’ and LVDD grading had similar predictive accuracy. Our findings support LVDD assessed by either E/e’ and LVDD as an independent risk factor for progression towards end-stage kidney disease. Because of the complexity of the interaction between cardiac and renal function, future prospective studies are warranted for confirmation and validation of our study findings.

## Data Availability

The data underlying this article cannot be shared publicly due to Danish law. The data will be shared on reasonable request to the corresponding author.
